# Outcomes and National Trends for the Surgical Treatment of Lumbar Spine Trauma

**DOI:** 10.1155/2016/3623875

**Published:** 2016-06-15

**Authors:** Doniel Drazin, Miriam Nuno, Faris Shweikeh, Alexander R. Vaccaro, Eli Baron, Terrence T. Kim, J. Patrick Johnson

**Affiliations:** ^1^Department of Neurosurgery, Cedars-Sinai Medical Center, Los Angeles, CA 90048, USA; ^2^Department of Surgery, University of Arizona College of Medicine, Tucson, AZ 85724, USA; ^3^Department of Orthopedic Surgery, Thomas Jefferson University, Philadelphia, PA 19107, USA; ^4^Department of Orthopedics, Cedars-Sinai Medical Center, Los Angeles, CA 90048, USA; ^5^Department of Neurosurgery, University of California Davis Medical Center, Sacramento, CA 95820, USA

## Abstract

*Introduction*. Operative treatment of lumbar spine compression fractures includes fusion and/or cement augmentation. Our aim was to evaluate postoperative differences in patients treated surgically with fusion, vertebroplasty, or kyphoplasty.* Methods*. The Nationwide Inpatient Sample Database search for adult vertebral compression fracture patients treated 2004–2011 identified 102,316 surgical patients: 30.6% underwent spinal fusion, 17.1% underwent kyphoplasty, and 49.9% underwent vertebroplasty. Univariate analysis of patient and hospital characteristics, by treatment, was performed. Multivariable analysis was used to determine factors associated with mortality, nonroutine discharge, complications, and patient safety.* Results*. Average patient age: fusion (46.2), kyphoplasty (78.5), vertebroplasty (76.7) (*p* < .0001). Gender, race, household income, hospital-specific characteristics, and insurance differences were found (*p* ≤ .001). Leading comorbidities were hypertension, osteoporosis, and diabetes. Risks for higher mortality (OR 2.0: CI: 1.6–2.5), nonroutine discharge (OR 1.6, CI: 1.6–1.7), complications (OR 1.1, CI: 1.0–1.1), and safety related events (OR 1.1, CI: 1.0–1.1) rose consistently with increasing age, particularly among fusion patients. Preexisting comorbidities and longer in-hospital length of stay were associated with increased odds of nonroutine discharge, complications, and patient safety.* Conclusions*. Fusion patients had higher rates of poorer outcomes compared to vertebroplasty and kyphoplasty cohorts. Mortality, nonroutine discharge, complications, and adverse events increased consistently with older age.

## 1. Introduction

Lumbar compression fractures are among the most common medical and surgical conditions encountered by spinal surgeons [1]. Approximately 1.4 million patients sustain vertebral compression fractures every year [2], with an annual inpatient cost just under $5 billion [3]. Compression fractures disproportionately affect the elderly (65+ years) secondary to osteoporosis, which is responsible for >700,000 spinal fracture cases in the United States annually [4]. Although traumatic lumbar fractures represent a small portion in all trauma patients, their physical and financial burden on patients are more significant than other injuries [5].

Standard treatment of vertebral compression fractures consists of conservative management, including bed rest, bracing, and analgesics [6]. Studies, however, have noted that these practices are often insufficient in improving pain and mobility of these patients [7–9]. Operative interventions, namely, surgical fusion with instrumentation and cement augmentation procedures, have been gaining popularity [10, 11] as studies have shown both short-term physical improvements [12–14] and long-term survival benefits [15, 16] in select patients undergoing surgical intervention for compression fractures.

Numerous studies have looked at national trends and outcomes of cement augmentation procedures (vertebroplasty and kyphoplasty) for vertebral compression fractures [13, 15–18]. These studies, however, do not evaluate differences in demographics and outcomes among surgical fusion, kyphoplasty, and vertebroplasty procedures performed for vertebral compression fractures. This study evaluates patient demographics and hospital characteristics associated with each type of treatment as well as assessing potential outcome differences between patients undergoing fusion, vertebroplasty, and kyphoplasty for vertebral compression fracture.

## 2. Methods

### 2.1. Data Source and Cohort Selection

We utilized the Nationwide Inpatient Sample (NIS) database to capture vertebral fracture patients that underwent vertebral cement augmentation procedures in US hospitals between 2004 and 2011. Using International Classification of Diseases-Ninth Revision-Clinical Modification (ICD-9-CM) codes, we identified adult patients 18 years of age or older with primary diagnosis of lumbar fracture (ICD-9-CM: 805.4, 806.4); only patients that underwent a fusion (ICD-9-CM: 81.0–81.08), kyphoplasty (ICD-9-CM: 81.65), or vertebroplasty (ICD-9-CM: 81.66) according to the five leading procedures documented were included. Patients who underwent spine augmentation for a vertebral fracture secondary to malignancy according to the leading five diagnoses of cancer (ICD-9, 1400–1991, 2000–2089) were excluded from this analysis.

### 2.2. Patient Population and Hospital Characteristics

Demographics considered included patients' age, gender, race, medical insurance, median income, and preexisting comorbidities. Hospital characteristics such as number of beds, teaching status, region, and location were documented. Age was analyzed as a continuous variable using the following categories: 18–44, 45–64, 65–84, and 85+. In terms of percentages, data was missing for gender (0.07%), race (21.9%), insurance (0.02%), median income (2.1%), hospital bed size (2.7%), teaching status (2.7%), and hospital location (2.7%).

### 2.3. Outcomes of Interest

In-hospital mortality, nonroutine discharge, complications, patient safety indicators, in-hospital length of stay (LOS), and total charges were considered in this study. A discharge other than discharge to home (e.g., transfer, mortality) was considered nonroutine. The following adverse events were considered in the overall complication rate: neurological, pulmonary, thromboembolic, cardiac, procedure related, medical, peripheral vascular, infection, fluid, and electrolyte abnormalities, cerebrospinal fluid rhinorrhea, stroke, and bleeding.

### 2.4. Statistical Analysis

Descriptive statistics were used to summarize patient and hospital characteristics. Bivariate analysis was used to determine differences in patient/hospital characteristics and outcomes according to the type of intervention experienced (e.g., cement augmentation and fusion). Odds ratio (OR), 95% confidence intervals (CI), interquartile range (IQR), standard deviation (SD), and corresponding *p* values were reported. US nationwide estimates were performed using SAS PROC SURVEY methodology. All analyses used SAS version 9.1 for Windows (SAS Institute Inc., Cary, NC). A *p* value ≤ .05 was considered statistically significant.

## 3. Results

### 3.1. Demographics

A total of 102,316 vertebral fracture patients underwent a cement augmentation or spinal fusion procedure between 2004 and 2011. A subset of 31,332 (30.6%) were treated with fusion, 17,456 (17.1%) underwent a kyphoplasty, 51,021 (49.9%) underwent a vertebroplasty, and the remaining 2,507 (2.5%) had multiple procedures (Table 1). Patients that underwent a fusion were significantly younger than the kyphoplasty and vertebroplasty cohorts, 46.2 versus 78.5 and 76.7, respectively (*p* < .0001). Only 1.6% of fusion patients were of age 85+ compared to significant rates found in kyphoplasty (33.1%) and vertebroplasty cohorts (27.6%). There were significantly fewer white patients in the fusion (79.5%) cohort compared to kyphoplasty (87.5%) and vertebroplasty cohorts (88.1%) (*p* < .0001). Significantly more fusion patients had private insurance (46.8%) compared to kyphoplasty (11.4%) and vertebroplasty cohorts (12.9%) (*p* < .0001). Differences according to median household income were also documented according to augmentation procedure type (*p* = .0002).

**Figure 1:**
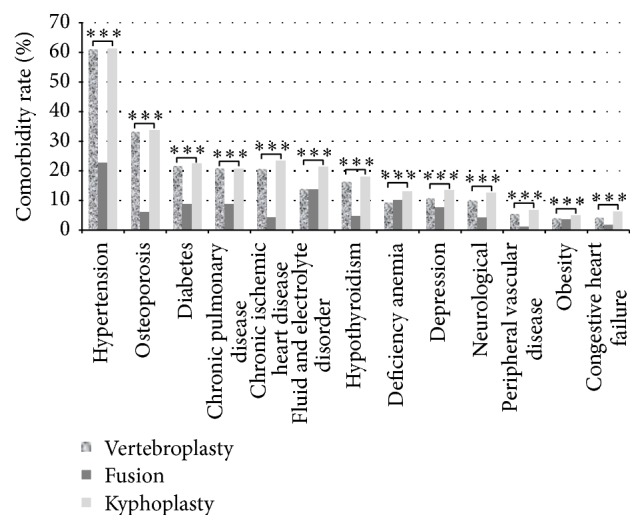
Distribution of comorbidities for vertebral compression fracture patients that underwent a fusion, kyphoplasty, or vertebroplasty between 2000 and 2011 (*N* = 102,316). ^*∗∗∗*^ *p* < .0001.

Hospital characteristics according to the type of augmentation procedure were also consistently significant (Table 2). More fusion patients were treated in large hospitals, teaching hospitals, and urban hospitals compared to the kyphoplasty, vertebroplasty, and multiple surgery cohorts (Table 2). A detailed description of preexisting comorbidities according to surgical procedure type is illustrated in Figure 1. Overall, hypertension followed by osteoporosis and diabetes seemed to be the most common conditions. Fusion patients had consistently and significantly (*p* < .0001) fewer comorbidities compared to kyphoplasty and vertebroplasty cohorts, independently of the type of comorbidity.

### 3.2. Univariate Analysis Outcomes

The overall mortality rate was 0.5%, with a leading rate of 0.8% among patients that underwent a fusion, 0.7% for kyphoplasty, 0.3% for vertebroplasty, and 1.4% for multiple procedures cohort (*p* < .0001, Table 3). Approximately half of all patients (55.4%) were discharged nonroutinely. Kyphoplasty patients were more likely to be discharged nonroutinely compared to fusion (51.7%) and vertebroplasty patients (52.7%) and patients that underwent multiple surgeries (64.3%). The highest rates of complications were documented among patients that underwent multiple surgeries (25.4%), followed by fusion patients (24.0%), kyphoplasty patients (21.4%), and vertebroplasty patients (16.0%). Similarly, fusion patients experienced significantly more adverse safety events (13.5%) when compared to kyphoplasty (8.8%), vertebroplasty (6.5%), and multiple procedures patients (12.0%) (*p* < .0001).

The average overall in-hospital length of stay was 6.5 days (Table 3). Patients that underwent a fusion spent a significantly (*p* < .0001) longer hospitalization period (9.8 days) compared to the multiple surgeries cohort (7.5 days), kyphoplasty cohort (5.8 days), and vertebroplasty cohort (4.6 days). Fusion patients spent an average of $113,067, while patients that underwent multiple procedures spent an average of $74,047 (*p* < .0001). Kyphoplasty ($34,363) and vertebroplasty ($42,459) patients had significantly lower total charges.

### 3.3. Multivariable Analysis of Patient Outcomes

After adjusting for patient's age, race, gender, type of insurance, preexisting comorbidities, and length of in-hospital stay, we found that older age was significantly associated with an increased risk of mortality (OR 2.0, CI: 1.6–2.5), nonroutine discharge (OR 1.6, CI: 1.6–1.7), complication (OR 1.1, CI: 1.0–1.1), and safety adverse events (OR 1.1, CI: 1.0–1.1) (Table 4). Increasing preexisting conditions and longer in-hospital length of stays were also significantly associated with higher odds of mortality, nonroutine discharge, complications, and safety related events (*p* < .0001). Patients that underwent a fusion procedure had significantly higher odds of mortality (OR 6.2, CI: 3.4–11.3), nonroutine discharge (OR 2.9, CI: 2.5–3.4), complication (OR 2.2, CI: 1.9–2.6), and safety related event (OR 1.8, CI: 1.5–2.2) compared to the vertebroplasty cohort. White patients seemed to have a significant increased risk of a complication (OR 1.2, CI; 1.0–1.4) and safety related event (OR 1.2: 1.0–1.5) compared to nonwhite patients.

### 3.4. Outcome Trends by Age and Surgical Procedure

**Figure 2:**
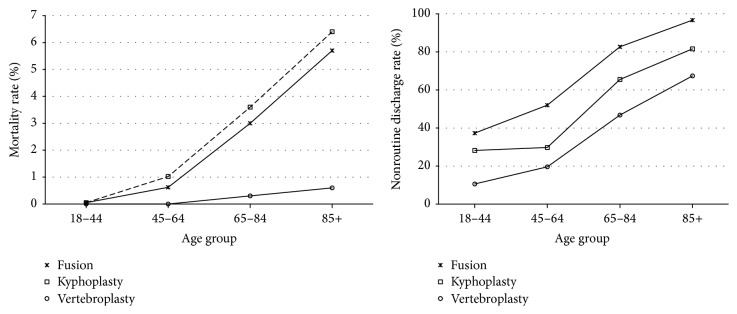
Mortality and nonroutine discharge rates by surgical procedure type and age (*N* = 102,316).

**Figure 3:**
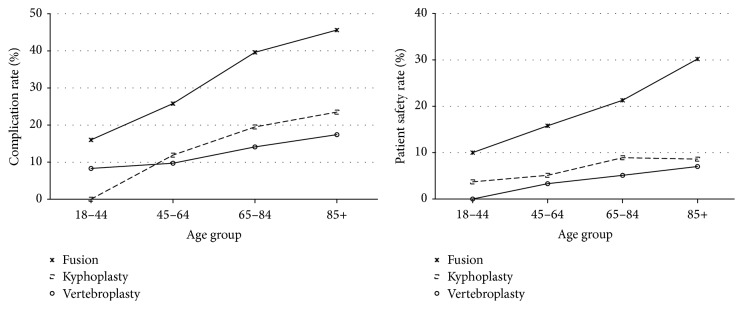
Complication and patient safety rates by surgical procedure type and age (*N* = 102,316).

 A consistently increasing trend in mortality as well as nonroutine discharge was observed among fusion, kyphoplasty, and vertebroplasty patients with older age (Figure 2). Patients that underwent a vertebroplasty had consistently higher mortality independently of age. Similarly, fusion patients had consistently higher rates of mortality independently of age. Fusion patients had higher nonroutine discharge rates compared to all cohorts independently of age. Fusion patients had significantly higher rates of safety indicators and complications compared to all other cohorts, independently of age (Figure 3).

## 4. Discussion

Lumbar compression fractures represent a leading cause of morbidity and mortality in the United States. Understanding the role of different surgical treatments in survival, complications, and other outcomes of interest is imperative in order to determine optimum treatment modalities. With increasing evidence supporting surgical intervention for these patients [12, 15, 16, 19–23], numerous studies have sought to compare outcomes among different treatment paradigms [6, 12, 15, 17, 24, 25]. Surgical fusion with instrumentation and cement augmentation have been of primary interest, as both procedures have seen increased utilization from the early 1990s to 2000s (12.9% for cement augmentation patients and 39% for surgical fusion) [26, 27]. Although numerous studies have compared outcomes between nonsurgical intervention, vertebroplasty, and kyphoplasty [12, 13, 15, 17, 18, 27], there have been no published studies comparing outcomes between vertebroplasty, kyphoplasty, and surgical fusion. The goal of our study was to evaluate differences in demographics and hospital characteristics as well as compare postsurgical outcomes between vertebroplasty, kyphoplasty, and fusion patients with the primary diagnosis of vertebral compression fractures.

Our findings suggest a slight female preponderance (58.8%) of patients undergoing surgery for lumbar spine trauma, with significantly more females receiving kyphoplasty (69.6%) and vertebroplasty (68.5%) compared to spinal fusion (37.0%). In the United States, the risk of developing a fragility fracture is up to 40% for women and 13% for men over the age of 50 [28]. More specifically, vertebral compression fractures occur in approximately 16% of all postmenopausal women [29]. In our cohort, 64.8% of lumbar trauma patients undergoing surgical intervention were over the age of 65. Significantly younger patients underwent surgical fusion (46.2 years) versus kyphoplasty (78.5 years) and vertebroplasty (76.7 years) procedures. This is consistent with an institutional study by Hsieh et al., which reported significantly older patients in their kyphoplasty versus short-segment fixation with I-VEP cohort [30]. Lad et al. reported that, within their cohort of vertebral spinal fracture patients, a majority were women and over the age of 65 [27]. As age and gender may be connected in this population, the explanation of higher percentages of elderly patients and women undergoing augmentation procedures may be related.

The most common mechanisms for the occurrence of lumbar spine trauma include falls, sport accidents, and motor vehicle crashes [31]. However, in elderly patients, osteoporosis is responsible for >700,000 vertebral compression fractures in the United States annually [4]. We found that osteoporosis was the second leading comorbidity for all lumbar spine trauma patients, with significantly higher percentages in cement augmentation versus surgical fusion cohorts. The leading comorbidity was hypertension, which is known to have high prevalence in elderly populations [32]. In 1990, the United States Census Bureau estimated that over 16 million Americans will be 85 years or older by the year 2050 [33]. Thus, the effect of age on outcomes and treatment for vertebral compression fracture patients is an important health-care problem of increasing impact. We found that, regardless of procedure, an increase in age is associated with higher nonroutine discharge rates, complication rates, and patient safety rates. Mortality rates for cement augmentation procedures remained relatively stable with increased age, while rates for fusion patients increased dramatically, especially in patients >65 years old.

Fusion patients as a whole had consistently higher mortality, complication, nonroutine discharge, and patient safety rates compared to their augmentation counterparts. There were also significantly more fusion procedures performed at large, urban teaching hospitals. Daniels et al. reported similar findings while evaluating hospital-based rates of thoracolumbar spine arthrodesis for patients with spinal fractures. They reported that hospitals with higher volumes of spinal fracture patients had higher fusion rates compared to hospitals treating fewer fracture patients [31]. This may indicate that fusion procedures are more complex in nature, which would explain the younger patients, lower comorbidities of patients, and increased adverse outcomes. Patients who underwent fusion were 6.2 times more likely to experience morality, 2.9 times more likely to have a nonroutine discharge, 2.2 times more likely to have a complication, and 1.8 times more likely to have PSI as compared to vertebroplasty patients. Similarly, kyphoplasty patients had higher percentages of adverse outcomes, such as a 70% increase in mortality, when compared to vertebroplasty patients. Chen et al. had contrary findings, which suggested that an adjusted risk of death was 20% lower for kyphoplasty patients versus vertebroplasty patients [15].

In a meta-analysis of literature, Eck et al. evaluated pain relief and risk of complications associated with kyphoplasty and vertebroplasty [25]. Their findings suggest that although both methods of augmentation are effective in relieving pain, there is a significant increase in pain relief for vertebroplasty patients versus kyphoplasty patients [12]. However, it was also reported that vertebroplasty patients had higher rates of complications compared to their kyphoplasty counterparts, including increased cement leakage and higher occurrence of sustaining subsequent vertebral fractures [34, 35]. These inconsistencies in results comparing vertebroplasty and kyphoplasty procedures and lack of studies comparing augmentation procedures to spinal fusion procedures call for further research. Prospective studies with appropriate matching of individuals undergoing each treatment type would address limitations of current studies and lead to more concrete results regarding the clinical outcomes of patients undergoing surgical intervention for lumbar spine trauma [15].

### 4.1. Limitations

Although utilization of national databases may be advantageous, namely, due to high volume of patients and avoidance of selection bias, there remain considerable limitations. Such limitations include potential coding errors, lack of data specifying type of injury and severity, and absence of long-term outcomes or disability scores [1, 26, 27]. Additionally, the codes and the coding used by physician may not be specific for compression fractures. Populations with operative fractures versus those with fractures treated with kyphoplasty/vertebroplasty are likely completely different with regard to injury severity, neurologic status, and so forth. Our goal in this study was to present the general trend in compression fractures in the elderly population over the years studied. Therefore, our findings should be considered with these points in mind.

## 5. Conclusions

Treatment patterns of patients with vertebral compression fractures differ significantly when comparing patient demographics and hospital type and location. Age and medical comorbidities are significant risk factors for mortality and adverse outcomes regardless of procedures. Spinal fusion was associated with higher risk of adverse outcomes compared to cement augmentation. Kyphoplasty was associated with higher risk of adverse outcome compared to vertebroplasty. Elderly patients and those with medical comorbidities appear to strongly have the potential for poorer outcomes regardless of what type of procedure is performed.

## Figures and Tables

**Table 1:** Characteristics of 102,316 vertebral compression fracture patients that underwent vertebral augmentation procedures between 2004 and 2011.

	All cases (*N* = 102,316)	Vertebral augmentation procedure type				*p* value^§^
		Fusion *N* = 31,332 (30.6)	Kyphoplasty *N* = 17,456 (17.1)	Vertebroplasty *N* = 51,021 (49.9)	Multiple surgeries *N* = 2,507 (2.5)	
Age in years						
Mean (SE)	67.5 (0.4)	46.2 (0.3)	78.5 (0.2)	76.7 (0.2)	68.3 (1.0)	<.0001
Median (IQR)	73 (54–82)	46 (31–58)	80 (72–86)	79 (70–85)	72 (57–81)	
Age by categories, *N* (%)						<.0001
18–44	15985 (15.6)	14772 (47.1)	212 (1.2)	725 (1.4)	276 (11.0)	
45–64	19963 (19.5)	11133 (35.5)	1730 (9.9)	6536 (12.8)	564 (22.5)	
65–84	45578 (44.5)	4912 (15.7)	9736 (55.8)	29687 (58.2)	1243 (49.6)	
85+	20791 (20.3)	516 (1.6)	5777 (33.1)	14074 (27.6)	424 (16.9)	
Female, *N* (%)	60155 (58.8)	11597 (37.0)	12149 (69.6)	34941 (68.5)	1469 (58.6)	<.0001
Race, *N* (%)						<.0001^*∗*^
White	71037 (85.4)	19560 (79.5)	12044 (87.5)	37651 (88.1)	1782 (87.8)	
Black	2220 (2.7)	962 (3.9)	355 (2.6)	848 (2.0)	54 (2.7)	
Hispanic	5448 (6.6)	2409 (9.8)	757 (5.5)	2176 (5.1)	106 (5.2)	
Asian/Pacific Islander	1886 (2.7)	620 (2.5)	343 (2.5)	891 (2.1)	32 (1.6)	
Native American	347 (0.4)	162 (0.7)	33 (0.2)	147 (0.3)	5 (0.2)	
Other	2195 (2.6)	882 (3.6)	233 (1.7)	1030 (2.4)	51 (2.5)	
Primary payer, *N* (%)						<.0001^*∗*^
Medicare	63622 (62.3)	5617 (18.0)	14642 (84.0)	41780 (82.0)	1584 (63.3)	
Medicaid	4462 (4.4)	3124 (10.0)	299 (1.7)	943 (1.9)	96 (3.8)	
Private insurance	23781 (23.3)	14600 (46.8)	1993 (11.4)	6509 (12.9)	590 (23.6)	
Self-pay	3973 (3.9)	3258 (10.4)	166 (1.0)	473 (0.9)	76 (3.0)	
No charge	321 (0.3)	237 (0.8)	10 (0.06)	70 (0.1)	5 (0.2)	
Others	5923 (5.8)	4354 (14.0)	324 (1.9)	1092 (2.1)	153 (6.1)	
Median income, *N* (%)						.0002
<$39,000	24614 (24.6)	8494 (28.0)	3685 (21.4)	11806 (23.6)	629 (25.8)	
$39,900–47,999	27666 (27.6)	8298 (27.3)	4825 (28.1)	13912 (27.8)	630 (25.9)	
$48,000–62,999	25572 (25.5)	7375 (24.3)	4357 (25.3)	13241 (26.4)	599 (24.5)	
>$63,000	22269 (22.20)	6207 (20.4)	4322 (25.1)	11160 (22.3)	580 (23.8)	

Rounded percent (%); missing data rates: female (.06), race (18.7), primary payer (.23), and median income (2.1).

^§^Comparisons between fusion, kyphoplasty, and vertebroplasty cohorts; ^*∗*^comparisons: white versus others and private versus nonprivate.

**Table 2:** Hospital characteristics of 102,316 vertebral compression fracture patients that underwent vertebral augmentation procedures between 2004 and 2011.

	All cases (*N* = 102,316)	Vertebral augmentation procedure type				*p* value^§^
		Fusion *N* = 31,332 (30.6)	Kyphoplasty *N* = 17,456 (17.1)	Vertebroplasty *N* = 51,021 (49.9)	Multiple surgeries *N* = 2,507 (2.5)	
Hospital bed size, *N* (%)						.001
Small	7442 (7.4)	1341 (4.3)	1539 (8.9)	4410 (8.7)	152 (6.2)	
Medium	24182 (23.9)	6928 (22.4)	4011 (23.1)	12580 (24.9)	664 (27.1)	
Large	69525 (68.7)	22617 (73.2)	11792 (68.0)	33482 (66.3)	1635 (66.7)	
Teaching hospital, *N* (%)						<.0001
Yes	49599 (49.0)	21819 (70.6)	7944 (45.8)	18595 (36.8)	1241 (50.6)	
No	51550 (51.0)	9066 (29.4)	9397 (54.2)	31877 (63.2)	1210 (49.4)	
Hospital region, *N* (%)						<.0001
Northeast	12556 (12.3)	4401 (14.0)	1666 (9.5)	6113 (12.0)	376 (15.0)	
Midwest	22374 (21.9)	6942 (22.2)	5025 (28.8)	9905 (19.4)	501 (20.0)	
South	49935 (48.8)	13428 (42.9)	7296 (41.8)	27996 (54.9)	1216 (48.5)	
West	17451 (17.1)	6561 (20.9)	3469 (19.9)	7007 (13.7)	414 (16.5)	
Hospital location, *N* (%)						.0003
Rural	6042 (6.0)	953 (3.1)	1182 (6.8)	3776 (7.5)	130 (5.3)	
Urban	95108 (94.0)	29932 (96.9)	16159 (93.2)	46696 (92.5)	2321 (94.7)	

Rounded percent (%); missing data rates: female (.06), race (18.7), primary payer (.23), median income (2.1), and hospital data (1.1).

^§^ Comparisons between fusion, kyphoplasty, and vertebroplasty cohorts.

**Table 3:** Outcomes of 102,316 vertebral compression fracture patients that underwent vertebral augmentation procedures between 2004 and 2011.

	All cases (*N* = 102,316)	Vertebral augmentation procedure type				*p* value^§^
		Fusion *N* = 31,332 (30.6)	Kyphoplasty *N* = 17,456 (17.1)	Vertebroplasty *N* = 51,021 (49.9)	Multiple surgeries *N* = 2,507 (2.5)	
Mortality rate, *N* (%)	560 (0.5)	246 (0.8)	115 (0.7)	164 (0.3)	35 (1.4)	<.0001
Nonroutine discharge, *N* (%)	56650 (55.4)	16184 (51.7)	11971 (68.6)	26883 (52.7)	1611 (64.3)	<.0001
Length of stay, days						<.0001
Average (SD)	6.5 (0.1)	9.8 (0.1)	5.8 (0.09)	4.6 (0.1)	7.5 (0.3)	
Median (IQR)	5 (2–8)	7 (5–11)	4 (3–7)	3 (1–6)	6 (3–10)	
Average total charges ($)						<.0001
Average (SD)	72304 (1774)	140484 (3707)	34363 (864)	42459 (796)	99293 (4656)	
Median (IQR)	45361 (26251–87838)	113067 (75839–167976)	26581 (17903–41173)	34898 (23504–51781)	74047 (41641–131571)	
Complications, *N* (%)						<.0001
Any	20091 (19.6)	7534 (24.0)	3743 (21.4)	8176 (16.0)	637 (25.4)	
0	8226 (80.4)	23798 (76.0)	13713 (78.6)	42845 (84.0)	1870 (74.6)	
1	17309 (16.9)	5793 (18.5)	3477 (19.9)	7518 (14.7)	522 (20.8)	
2	2339 (2.3)	1424 (4.5)	244 (1.4)	593 (1.2)	78 (3.1)	
3+	442 (0.4)	316 (1.0)	23 (0.1)	65 (0.1)	38 (1.5)	
Patient safety indicators, *N* (%)^*∗*^						
Any	9406 (9.2)	4235 (13.5)	1537 (8.8)	3334 (6.5)	300 (12.0)	<.0001
0	92910 (90.8)	27097 (86.5)	15919 (91.2)	47687 (93.5)	2207 (88.0)	
1	8107 (7.9)	3444 (11.0)	1365 (7.8)	3047 (6.0)	251 (10.0)	
2	1015 (1.0)	627 (21.0)	118 (0.7)	231 (0.5)	40 (1.6)	
3+	286 (0.3)	164 (0.5)	55 (0.3)	57 (0.1)	9 (0.4)	

Rounded percent (%).

^§^Comparisons between fusion, kyphoplasty, and vertebroplasty cohorts.

**Table 4:** Adjusted odds ratio (OR) and 95% confidence intervals (CI) for factors associated with mortality, nonroutine discharge, complication, and safety related adverse events (*N* = 102,316).

	Outcomes							*p* value
	Mortality, OR (95% CI)	*p* value	Nonroutine discharge OR (95% CI)	*p* value	Complication OR (95% CI)	*p* value	Patient safety indicators OR (95% CI)	
Age in decades	2.0 (1.6–2.5)	<.0001	1.6 (1.6–1.7)	<.0001	1.1 (1.0–1.1)	.002	1.1 (1.0–1.1)	<.0001
Gender (ref: male)								
Female	0.7 (0.4–1.1)	.14	1.2 (1.1–1.3)	<.0001	1.0 (0.9–1.1)	.40	0.7 (0.6–0.8)	<.0001
Race (ref: nonwhite)								
White	1.3 (0.6–1.6)	.51	1.1 (1.0–1.3)	.08	1.2 (1.0–1.4)	.02	1.2 (1.0–1.5)	.03
Insurance (ref: nonprivate)								
Private	0.8 (0.4–1.6)	.54	1.0 (0.9–1.1)	.79	1.0 (0.9–1.1)	.91	1.1 (0.9–1.3)	.26
Comorbidities^*∗*^	1.1 (0.9–1.2)		1.3 (1.3-1.3)	<.0001	1.6 (1.6–1.7)	<.0001	1.3 (1.2–1.4)	<.0001
Type of procedure (ref: vertebroplasty)								
Fusion	6.2 (3.4–11.3)	<.0001	2.9 (2.5–3.4)	<.0001	2.2 (1.9–2.6)	<.0001	1.8 (1.5–2.2)	<.0001
Kyphoplasty	1.7 (1.0–3.0)	.04	1.5 (1.3–1.7)	<.0001	1.2 (1.1–1.4)	.003	1.1 (0.9–1.3)	.19
Length of stay, days	1.1 (1.1-1.1)	<.0001	1.2 (1.2-1.2)	<.0001	1.1 (1.1-1.1)	<.0001	1.1 (1.1-1.1)	<.0001

Outcome analysis excludes patients that had multiple procedures (*N* = 1,856, 2.5%).

^*∗*^Unit increments.
